# Bartter Syndrome Type 1 Due to Novel *SLC12A1* Mutations Associated With Pseudohypoparathyroidism Type II

**DOI:** 10.1210/jcemcr/luad019

**Published:** 2023-02-24

**Authors:** Zentaro Kiuchi, Kandai Nozu, Kunimasa Yan, Harald Jüppner

**Affiliations:** Department of Pediatrics, Kyorin University School of Medicine, Mitaka, Tokyo, Japan; Department of Pediatrics, Kobe University Graduate School of Medicine, Kobe, Hyogo, Japan; Department of Pediatrics, Kyorin University School of Medicine, Mitaka, Tokyo, Japan; Department of Pediatrics, Kosei Hospital, Suginami, Tokyo, Japan; Endocrine Unit, Massachusetts General Hospital and Harvard Medical School, Boston, MA, USA; Pediatric Nephrology Unit, Massachusetts General Hospital and Harvard Medical School, Boston, MA, USA

**Keywords:** Bartter syndrome, *SLC12A1*, hyperparathyroidism, Ellsworth Howard test, pseudohypoparathyroidism type II, NSAID

## Abstract

Bartter syndrome type 1 is caused by mutations in the solute carrier family 12 member 1 (*SLC12A1*), encoding the sodium-potassium-chloride cotransporter-2 (NKCC2). In addition to causing renal salt-losing tubulopathy, *SLC12A1* mutations are known to cause nephrocalcinosis due to hypercalciuria, as well as failure to thrive associated with abnormal calcium and phosphorus homeostasis. We report a now 7-year-old Japanese girl with polyuria, hyponatremia, hypokalemia, and metabolic alkalosis, in whom compound heterozygous novel *SLC12A1* mutations were identified. Elevated parathyroid hormone (PTH) levels were consistently noted after the age of 1 year in conjunction with gradually declining serum calcium and increasing serum phosphorus levels. To confirm suspected PTH-resistance, Ellsworth Howard tests were performed at the ages of 6 years 8 months and 6 years 10 months in the absence or presence of ibuprofen, respectively. Urinary adenosine 3′,5′-cyclic monophosphate excretion increased on both occasions in response to PTH(1-34) infusion suggesting pseudohypoparathyroidism type II. However, only during treatment with ibuprofen did PTH induce an almost normal phosphaturic response. The nonsteroidal anti-inflammatory drugs thus enhanced growth velocity, alleviated hypercalciuria, and increased PTH-stimulated urinary phosphorus excretion without significantly affecting renal function.

Bartter syndrome (BS) type 1 is a rare renal salt-losing tubulopathy with an autosomal recessive mode of inheritance, which is caused by homozygous or compound heterozygous mutations in the solute carrier family 12 member 1 (*SLC12A1*). *SLC12A1* encodes the sodium-potassium-chloride cotransporter-2 (NKCC2), which is expressed in the thick ascending limb (TAL) of the loop of Henle, where it plays a central role in renal salt reabsorption [1]. Patients affected by antenatal BS type 1 are typically born prematurely due to polyhydramnios and develop polyuria after birth. Especially during the neonatal period, life-threatening events such as dehydration, hyponatremia, and hypokalemia may develop, in addition to metabolic alkalosis, hyperreninemic hyperaldosteronism, failure to thrive, and osteopenia. Furthermore, hypercalciuria may lead to nephrocalcinosis and possibly impaired renal function [2]. Although BS type 1 is known to cause renal calcifications, osteopenia, and abnormal growth, the mechanisms leading to the associated impaired calcium and phosphorus homeostasis remain unclear.

## Case Presentation

**Figure 1.**
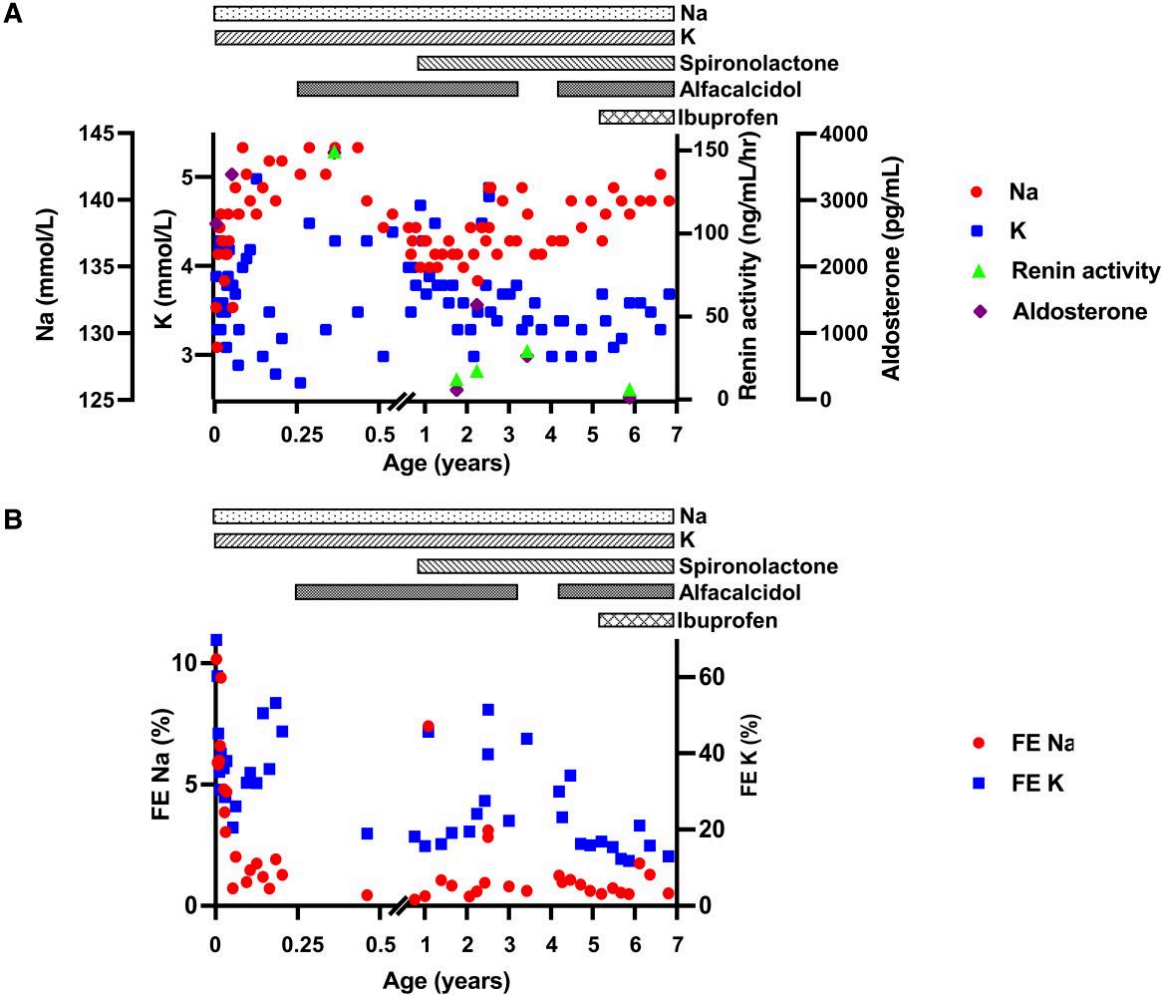
(A) Time-course of serum Na, K, renin activity, and aldosterone levels. Serum sodium and potassium were maintained within the normal ranges with sodium and potassium supplementations. Ibuprofen decreased renin and aldosterone levels. (B) Time-course of FE Na and K (FE Na and FE K). Urinary excretion of sodium and potassium gradually decreased thus allowing the decrease in the amount of sodium and potassium supplementations; however, these could not be discontinued. Abbreviations: FE, fractional excretion; K, potassium; Na, sodium.

This case is a currently 7-year-old Japanese girl. At 24 weeks of gestation, polyhydramnios was noticed. She was born by vaginal delivery at 33 weeks 3 days with a birth weight of 1907 g (appropriate for gestational age). She was admitted to the neonatal intensive care unit due to neonatal asphyxia (Apgar scores of 2 at 1 minute, 8 at 5 minutes). Polyuria was observed from day 1 of life that was accompanied by mild metabolic alkalosis, and hyponatremia and hypokalemia were noticed on day 2 (Fig. 1A) in association with increased urinary electrolyte excretion. Her aldosterone level was elevated (Fig. 1A), but she had no hypertension and no edema. Urine osmolality was consistently low. Both kidneys revealed no abnormalities upon ultrasonographic examination. She was discharged from the neonatal intensive care unit on day 77.

**Figure 2.**
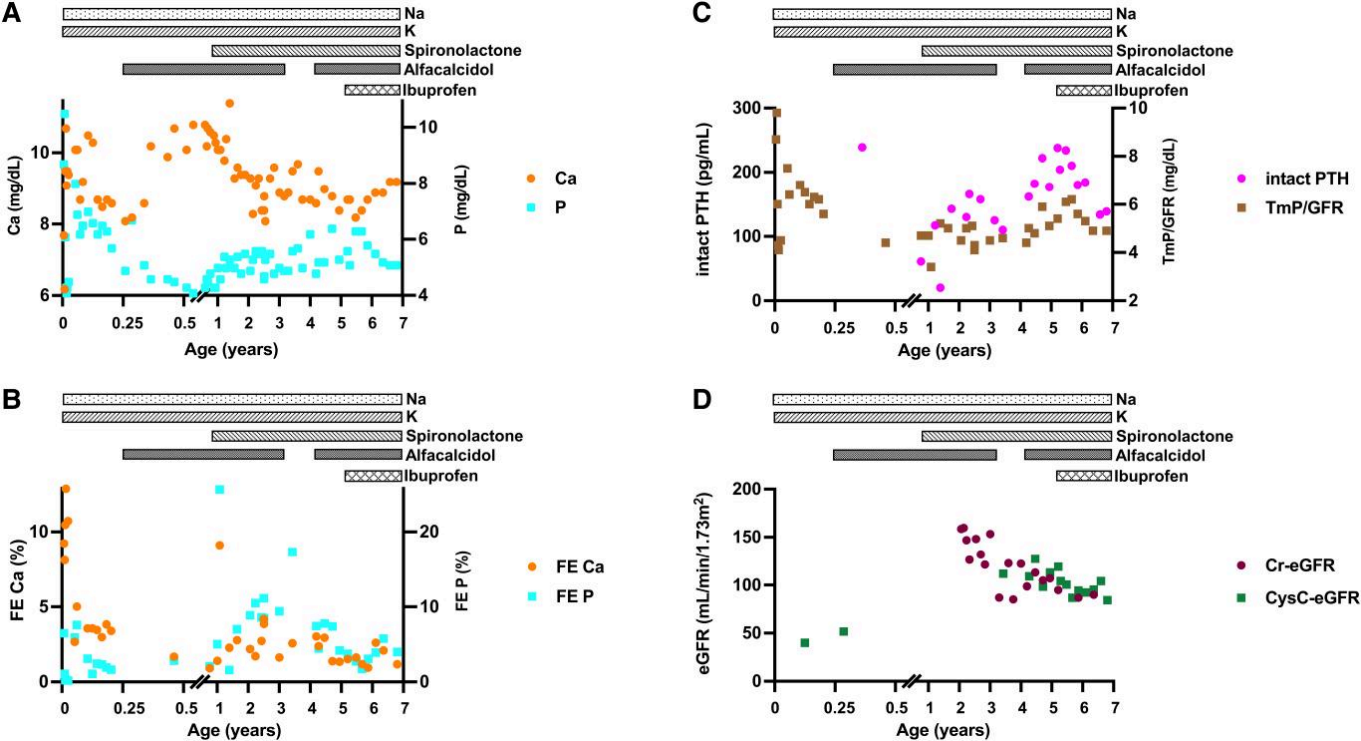
(A) Time-course of serum Ca and P levels. Our patient had hypocalcemia and hyperphosphatemia after the age of 2 years. Daily administration of ibuprofen was associated with improvements of both measurements. (B) Time-course of FE Ca and P (FE Ca and FE P). Ibuprofen was effective in suppressing hypercalciuria. (C) Time-course of serum intact PTH levels and TmP/GFR. Elevated PTH levels were observed after the age of 1 year (117 pg/mL), which subsequently increased gradually and remained abnormally elevated until the age of 5 years. Administration of ibuprofen appeared to decrease, but did not normalize PTH levels. Her TmP/GFR remained elevated. (D) Time-course of eGFR based on Cr or CysC measurements. A slight decrease in eGFR was observed after starting the administration of ibuprofen, but renal function remained stable subsequently. Abbreviations: Ca, calcium; Cr, creatinine; CysC, cystatin C; eGFR, estimated glomerular filtration rate; FE, fractional excretion; P, phosphorus; TmP/GFR, tubular reabsorption of phosphate per glomerular filtration rate.

Her psychomotor development has been within normal limits, but she failed to thrive, and echogenicity of the renal medulla was apparent at 5 months. During infancy, intact PTH levels were initially normal (normal 10-65 pg/mL) but then increased gradually and in association with mildly reduced serum calcium levels (2.2 mmol/L (8.7 mg/dL); normal 2.3-2.5 mmol/L (9.0-10.1 mg/dL), elevated serum phosphorus levels (2.0 mmol/L (6.2 mg/dL); normal 1.3-1.7 mmol/L (3.9-5.3 mg/dL) at 4 years, and maximal tubular reabsorption of phosphate per glomerular filtration rate (TmP/GFR) that was consistently at the upper end of normal (normal 3.8-5.0 mg/dL), ie, inappropriate for the elevated serum phosphate levels (Fig. 2A and 2C). Taken together, these data were thought to indicate PTH-resistance and suggested that NKCC2, which is primarily expressed in the TAL, could be involved, directly or indirectly, in mediating the actions of PTH at the PTH/PTHrP receptor. A neck ultrasound provided no evidence for enlarged parathyroid glands. Her estimated GFR (eGFR) was more than 85 mL/min/1.73 m^2^ (normal 84-157 mL/min/1.73 m^2^) (Fig. 2D), thus excluding chronic kidney disease as the cause of the elevated PTH levels.

## Diagnostic Assessment

**Figure 3.**
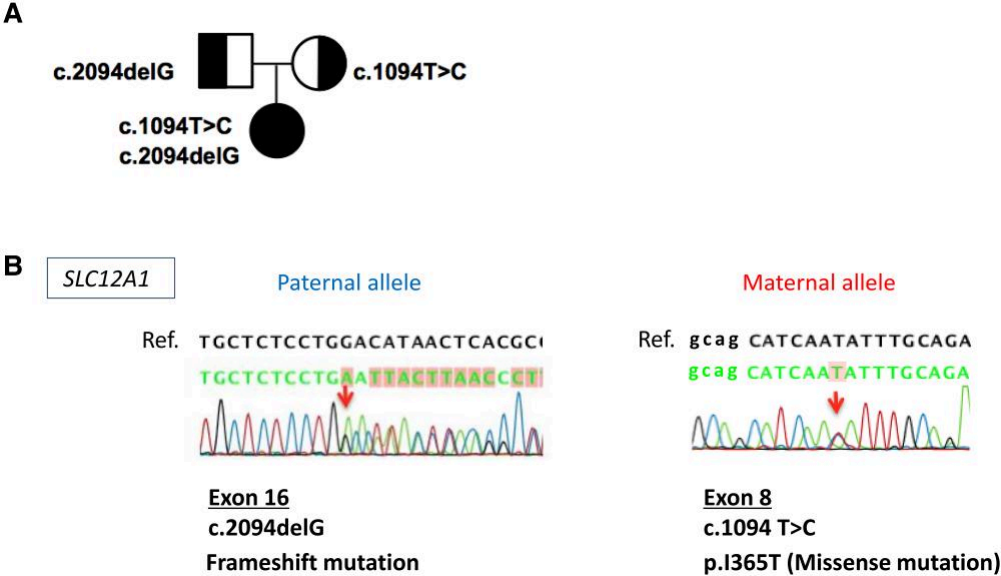
(A) Pedigree of the studied family and the identified *SLC12A1* mutations. (B) Sanger sequencing revealed for patient compound heterozygosity for 2 novel *SLC12A1* mutations, c.2094delG in exon 16 inherited from the father and c.1094T > C (p.I365T) in exon 8 inherited from the mother.

Based on clinical symptoms and laboratory findings, antenatal BS was suspected. Genetic analysis at the age of 10 months revealed compound heterozygous *SLC12A1* mutations, a novel c.2094delG in exon 16 inherited from the father and a novel c.1094T > C (p.I365T) in exon 8 inherited from the mother, thus confirming the diagnosis of BS type 1 (Fig. 3A and 3B).

## Treatment

The patient had neonatal asphyxia after delivery and needed endotracheal intubation, fluid resuscitation, supplemental sodium (starting dose: 5.6 mEq/kg/day, recently: 1.4 mEq/kg/day), and potassium (starting dose: 6.5 mEq/kg/day, recently: 1.3 mEq/kg/day) (Fig. 1A). These supplements were gradually decreased as urinary losses of sodium and potassium declined (Fig. 1B) but could not be discontinued because of persistently impaired renal tubular function. During the first months of life, she had low serum calcium, along with reduce serum phosphate and elevated alkaline phosphatase (ALP) levels. The patient was therefore started on alfacalcidol (starting dose: 0.04 µg/kg/day, recently: 0.03 µg/kg/day) to prevent the development of rickets. At 10 months, spironolactone (starting: 1.7 mg/kg/day, recently: 1.0 mg/kg/day) was initiated to maintain serum normal potassium levels. Because hypercalciuria and renal calcifications worsened, alfacalcidol was temporarily suspended at the age of 3 years 5 months. However, by the age of 4 years PTH levels had increased persistently with calcium concentrations at the lower end of normal. Alfacalcidol was therefore restarted to normalize serum calcium levels and to thereby suppress PTH secretion.

**Figure 4.**
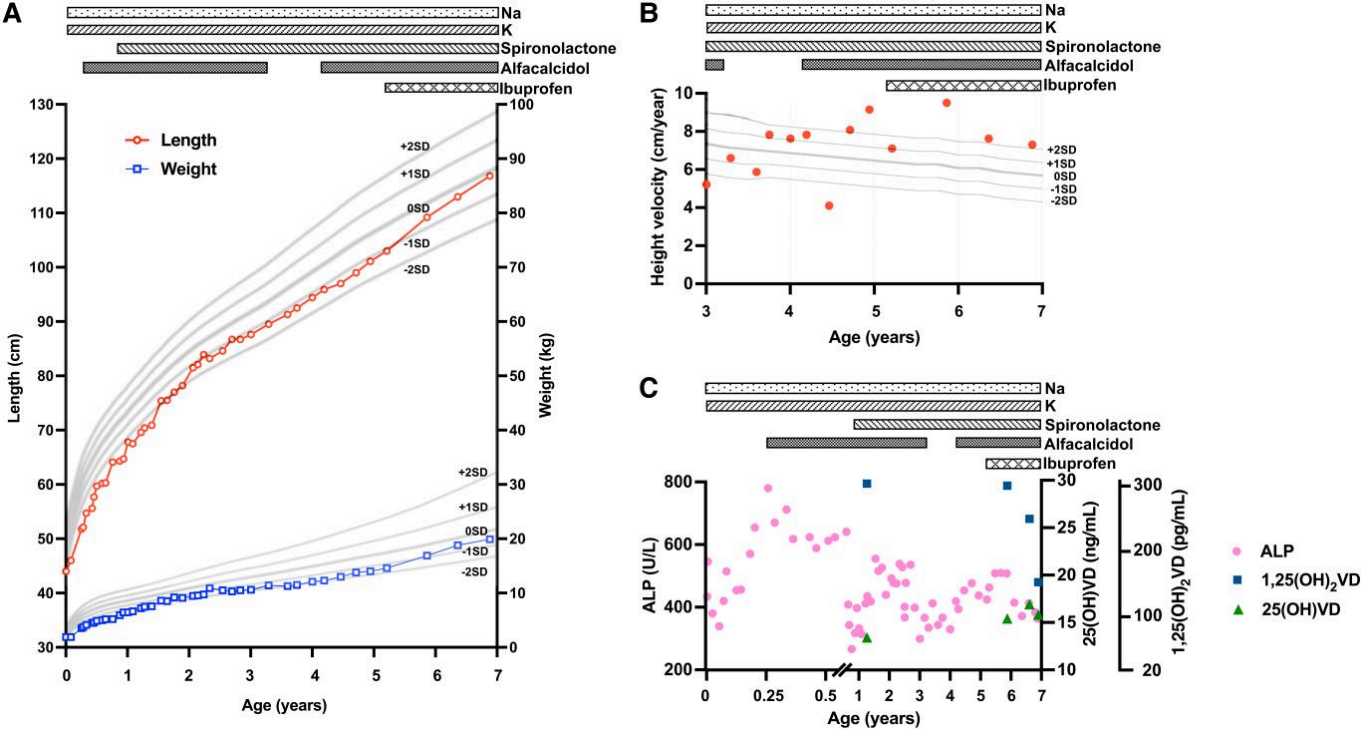
(A) The growth chart for Japanese girls. Body length and weight were approximately 2SD below average (2.3th percentile), which improved once serum electrolytes were normalized. After starting treatment with ibuprofen, growth velocity improved further. (B) Growth velocity chart between the ages of 3 and 7 years. Growth velocity increased after starting treatment with ibuprofen after the age of 5 years. (C) Time-course of ALP, 25(OH)VD, and 1,25(OH)_2_VD. ALP gradually decreased by the age of 6 years after treatment had been started with ibuprofen, which inhibits the cyclooxygenase enzyme thereby reducing levels of PGE_2_ that stimulates 1α-hydroxylation of 25(OH)VD in the kidney. Abbreviations: 25(OH)VD, 25-hydroxyvitamin D; 1,25(OH)_2_VD, 1,25-dihydroxyvitamin D; ALP, alkaline phosphatase; PGE_2_, prostaglandin E_2_.

In BS type 1, chloride ions are not reabsorbed via the TAL into the macula densa, thus activating cyclooxygenase, which increases prostaglandin E_2_ (PGE_2_) production thereby enhancing renin and aldosterone secretion [1]. Elevated PGE_2_ levels furthermore augment 1,25-dihydroxyvitamin D (1,25(OH)_2_VD) synthesis, thus contributing to hypercalciuria in BS [2]. In fact, in our patient the 1,25(OH)_2_VD level had been elevated to 305 pg/mL (normal 20-70 pg/mL) (Fig. 4C) but declined after initiating ibuprofen treatment (starting dose: 15 mg/kg/day, recently: 20 mg/kg/day) at the age of 5 years 2 months.

## Outcome and Follow-up

Body length and weight had been at least 2SD below average (2.3th percentile) for the first 2 years of life (Fig. 4A) but improved once serum electrolytes were consistently within normal limits (Figs. 1A and 2A). After ibuprofen treatment was initiated, growth velocity increased further (Fig. 4B), which is consistent with previous reports showing that administration of nonsteroidal anti-inflammatory drugs (NSAIDs) enhances growth in BS type 1 [3]. At the age of 6 years 7 months, anterior pituitary hormones were within normal limits (Table 1), the patient's bone age was consistent with her chronological age, and there was no radiographic evidence for rickets. Furthermore, the fourth and fifth metacarpals were normal in length, and there was no clinical evidence for Albright hereditary osteodystrophy. Renal function declined slightly after starting treatment with ibuprofen, but the eGFR has subsequently remained above 85 mL/min/1.73 m^2^ (Fig. 2D).

**Table 1.** Endocrinological test results at the age of 6 years 7 months, which revealed no abnormalities for anterior pituitary hormones

Endocrinological test	Results	Reference ranges
TSH	2.1 mIU/L (2.1 μIU/mL)	0.7-6.4 mIU/L (0.7-6.4 μIU/mL)
FT_3_	6.0 pmol/L (3.9 pg/mL)	3.7-8.6 pmol/L (2.4-5.6 pg/mL)
FT_4_	15 pmol/L (1.2 ng/dL)	10-28 pmol/L (0.8-2.2 ng/dL)
GH	1.0 μg/L (1.0 ng/mL)	0.7-6.0 μg/L (0.7-6.0 ng/mL)
IGF-I	32 nmol/L (243 ng/mL)	11-34 nmol/L (82-262 ng/mL)
LH	<0.20 IU/L (<0.2 mIU/mL)	0.02-0.18 IU/L (0.02-0.18 mIU/mL)
FSH	1.4 IU/L (1.4 mIU/mL)	1.0-4.2 IU/L (1.0-4.2 mIU/mL)
E_2_	<88 pmol/L (<24 pg/mL)	18-73 pmol/L (5.0-20 pg/mL)
ACTH	1.8 pmol/L (8.2 pg/mL)	1.7-4.9 pmol/L (7.7-22.1 pg/mL)
Cortisol	204 nmol/L (7.4 μg/dL)	166-607 nmol/L (6.0-22 μg/dL)
DHEA-S	0.24 μmol/L (9.0 μg/dL)	0.51-3.90 μmol/L (19-144 μg/dL)
Prolactin	3.6 μg/L (3.6 ng/mL)	3.0-24 μg/L (3.0-24 ng/mL)
ADH	14.4 pmol/L (15.6 pg/mL)	3.7-11.1 pmol/L (4.0-12 pg/mL)

ADH secretion was increased, possibly to compensate for the concentrating defect due to impaired renal tubular function in Bartter syndrome.

Abbreviations: ADH, antidiuretic hormone; DHEA-S, dehydroepiandrosterone sulfate; E_2_, estradiol; FT_3_, free triiodothyronine; FT_4_, free thyroxine; GH, growth hormone; IGF-I, insulin like growth factor-1; LH, luteinizing hormone.

Other NSAIDs, such as indomethacin, were reported to inhibit cyclooxygenase activity, thereby blocking excess synthesis of PGE_2_, which is known to stimulate 1α-hydroxylation of 25-hydroxyvitamin D (25(OH)VD) by the kidney [2]. In fact, we showed that the urinary excretion of PGE-MUM, a stable PGE_2_ metabolite, was elevated to 56.8 μg/g creatinine (Cr) (normal: <17 μg/g Cr) in the absence of an NSAIDs, while treatment with ibuprofen (20 mg/kg/day) was associated with lower PGE-MUM excretion (39.1 μg/g Cr). Reduced 1,25(OH)_2_VD levels (Fig. 4C) probably limited intestinal calcium absorption therefore lowering urinary calcium excretion (Fig. 2B) [2]. The increased serum calcium levels, which decreased serum PTH and surprisingly phosphate levels therefore most likely involved an 1,25(OH)_2_VD-independent mechanism (Fig. 2A and 2C). However, long-term observation will be needed to determine whether reduced urinary calcium excretion can stabilize or even improve renal calcifications, thus helping to preserve renal function. Bone mineral density, assessed 7 months and 20 months after initiating ibuprofen administration, increased from 0.53 g/cm^2^ to 0.66 g/cm^2^ (normal: 0.55-0.70 g/cm^2^ at 6 years), indicating that NSAIDs may have prevented progression to osteopenia.

**Figure 5.**
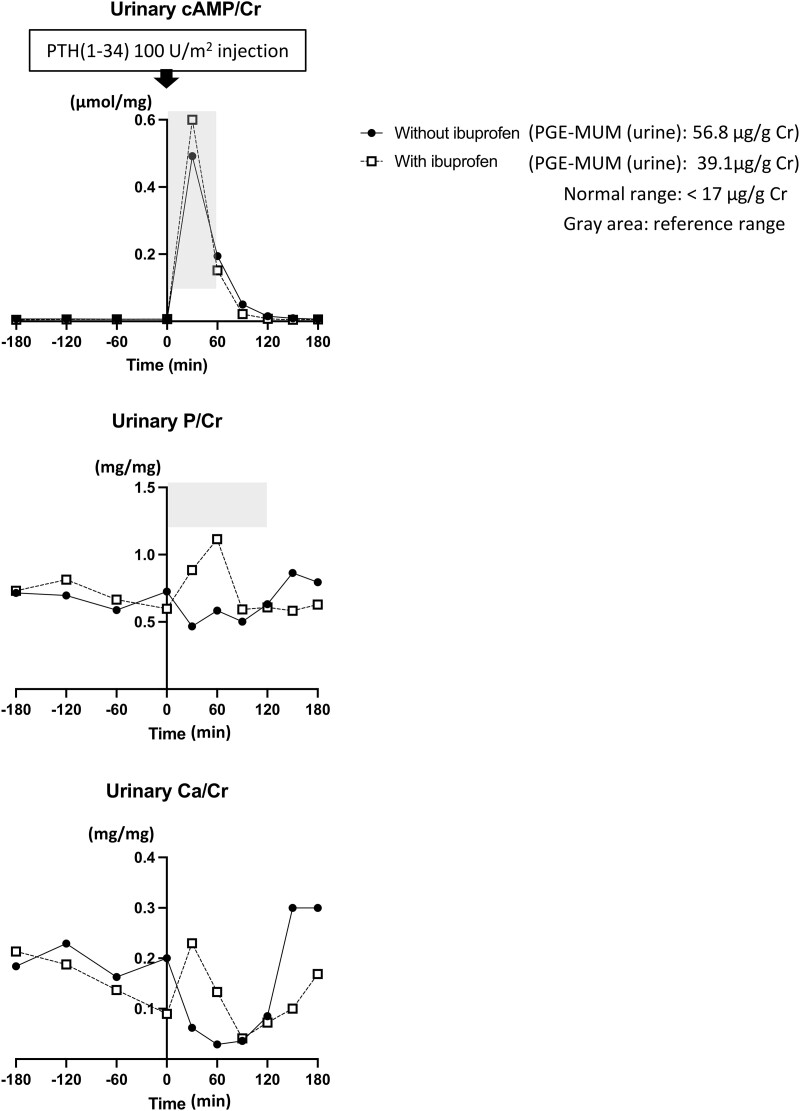
The results of the modified Ellsworth Howard test to determine whether the proximal renal tubules respond to PTH with an appropriate increase in urinary phosphate and cAMP excretion. After ensuring appropriate hydration of the patient, three 60-minute urine collections were obtained through a Foley catheter for baseline measurements. Subsequently, synthetic human PTH(1-34) (100 U/m^2^ (30 μg/m^2^)) was administered i.v. over 3 minutes and urine samples were then collected every 30 minutes over 3 hours. Phosphorus, calcium, and cAMP, as well as creatinine, were measured in each urine sample. A PTH-dependent increase of urinary phosphate excretion by more than 35 mg/2 hours over baseline is considered to be normal. An increase in urinary cAMP excretion of more than 1 μmol/hour or more than 10 times above basal is considered to be a good response to PTH(1-34) administration. Ellis Howard tests were performed in the absence or presence of ibuprofen (20 mg/kg/day). The PGE_2_ metabolite PGE-MUM was significantly elevated in absence of ibuprofen and lower, but not normal, when the patient was treated with the NSAIDs. In both tests, urinary cAMP excretion increased appropriately after PTH(1-34) administration. However, in the absence of ibuprofen, PTH failed to increase urinary phosphorus excretion. The patient was therefore diagnosed with pseudohypoparathyroidism type II. In the presence of ibuprofen, PTH(1-34) increased urinary phosphorus excretion robustly. The urinary calcium excretion was reduced in response to PTH(1-34) administration, indicating preserved distal tubular actions of PTH. Abbreviations: NSAID, nonsteroidal anti-inflammatory drug; PGE_2_, prostaglandin E_2_.

PTH normally increases urinary phosphorus excretion by reducing expression of the sodium-dependent phosphate transporters NPT2a and NPT2c in the renal proximal tubules. Our patient had elevated PTH levels before the initiation of Ibuprofen treatment, yet TmP/GFR was above the normal range, despite increased serum phosphate levels (Fig. 2C). This suggested PTH-resistance and Ellsworth Howard (EH) tests, modified to measure not only urinary phosphate but also adenosine 3′,5′-cyclic monophosphate (cAMP) excretion, were therefore performed at the ages of 6 years 8 months and 6 years 10 months either without or with ibuprofen treatment, respectively; alfacalcidol had been discontinued for 1 week prior to each test. Both EH tests revealed normal urinary cAMP excretion in response to PTH(1-34) administration (Fig. 5, upper panel). In the absence of Ibuprofen, there was no PTH-induced urinary phosphorus excretion, which is consistent with pseudohypoparathyroidism (PHP) type II (Fig. 5, middle panel). However, phosphorus excretion in response to PTH(1-34) approached the reference range during NSAIDs treatment. Urinary calcium excretion was decreased by PTH(1-34) in both tests, indicating a normal response to the hormone in the distal renal tubules (Fig. 5, lower panel).

## Discussion

We described a patient with BS type 1 due to novel *SLC12A1* compound heterozygous mutations, who developed during infancy mild hypocalcemia and hyperphosphatemia despite elevated PTH levels. Recent studies showed that approximately half of the investigated BS patients presented with secondary hyperparathyroidism [4], but the mechanism underlying the abnormal mineral ion regulation has remained unclear. For our patient, infusion of PTH(1-34) revealed strong evidence for PHP type II, ie, PTH-induced excretion of cAMP, but not phosphate, at least in the absence of ibuprofen treatment. This rare variant of PHP can be associated with acrodysostosis and resistance to multiple hormones, if caused by *PRKAR1A* mutations [5]. Bando et al furthermore suggested that hypokalemia may have contributed in a BS patient to the development PTH-resistance, as documented by performing an EH test before and after correction of serum potassium levels [6]. PHP type II has also been reported to be associated with calcium or vitamin D deficiency, but the underlying mechanisms remain unresolved [7, 8]. Elevated PGE_2_ levels, which are known to cause of hypercalciuria, nephrocalcinosis, and osteopenia in BS type 1, have not yet been implicated in the pathogenesis of PTH resistance. However, earlier studies had shown that PGE_2_ antagonizes the phosphaturic actions of PTH but not those of 8-bromo cAMP [9, 10]. Interestingly, the findings in our case are consistent with this observation, and therefore it is conceivable that NSAIDs treatment improves PTH responsiveness if NKCC2 protein is mutated, thus potentially implicating increased PGE_2_ levels in the development of renal PTH resistance.

In conclusion, our case with BS type 1 presented after the age of 2 years with hypocalcemia, hyperphosphatemia, and secondary hyperparathyroidism, and the EH test revealed PHP type II. Treatment with ibuprofen not only improved growth velocity and reduced urinary calcium excretion but also increased urinary phosphorus excretion in response to PTH(1-34) administration. We speculate that increased PGE_2_ levels may contribute, directly or indirectly, to the development of PTH resistance in the proximal renal tubules.

## Learning Points

- In antenatal BS type 1, NSAIDs administration reduces urinary calcium excretion, improves growth velocity, and may help prevent osteopenia.
- EH-testing using PTH(1-34) provided evidence for PHP type II in our BS type 1 case, who had developed hypocalcemia, hyperphosphatemia, and hyperparathyroidism during infancy.
- Ibuprofen increased urinary phosphorus excretion in response to PTH(1-34) administration and reduced the levels of PGE_2_, thus potentially implicating this prostaglandin in the development of PTH-resistance in BS type 1.

## Data Availability

Original data generated and analyzed for this report are included in this published article.
